# Expression of TRAIL-splice variants in gastric carcinomas: identification of TRAIL-γ as a prognostic marker

**DOI:** 10.1186/1471-2407-13-384

**Published:** 2013-08-12

**Authors:** Andreas Krieg, Sabrina Mersch, Nadine Wolf, Nikolas H Stoecklein, Pablo E Verde, Jan Schulte am Esch, Sebastian Heikaus, Helmut E Gabbert, Wolfram T Knoefel, Csaba Mahotka

**Affiliations:** 1 Department of Surgery A, Heinrich Heine University and University Hospital Duesseldorf, Moorenstrasse 5, 40225 Duesseldorf, Germany; 2 Institute of Pathology, Heinrich Heine University and University Hospital Duesseldorf, Moorenstrasse 5, 40225 Duesseldorf, Germany; 3 Coordination Centre for Clinical Trials, Heinrich Heine University and University Hospital Duesseldorf, Moorenstrasse 5, 40225 Duesseldorf, Germany

**Keywords:** TRAIL, Alternative splicing, Apoptosis, Gastric carcinoma

## Abstract

**Background:**

TNF-related apoptosis inducing ligand (TRAIL) belongs to the TNF-superfamily that induces apoptotic cell death in a wide range of neoplastic cells *in vivo* as well as *in vitro*. We identified two alternative TRAIL-splice variants, i.e. TRAIL-β and TRAIL-γ that are characterized by the loss of their proapoptotic properties. Herein, we investigated the expression and the prognostic values of the TRAIL-splice variants in gastric carcinomas.

**Methods:**

Real time PCR for amplification of the TRAIL-splice variants was performed in tumour tissue specimens and corresponding normal tissues of 41 consecutive patients with gastric carcinoma. Differences on mRNA-expression levels of the TRAIL-isoforms were compared to histo-pathological variables and correlated with survival data.

**Results:**

All three TRAIL-splice variants could be detected in both non-malignant and malignant tissues, irrespective of their histological staging, grading or tumour types. However, TRAIL-β exhibited a higher expression in normal gastric tissue. The proapoptotic TRAIL-α expression was increased in gastric carcinomas when compared to TRAIL-β and TRAIL-γ. In addition, overexpression of TRAIL-γ was associated with a significant higher survival rate.

**Conclusions:**

This is the first study that investigated the expression of TRAIL-splice variants in gastric carcinoma tissue samples. Thus, we provide first data that indicate a prognostic value for TRAIL-γ overexpression in this tumour entity.

## Background

TRAIL/APO2L (tumour necrosis factor related apoptosis-inducing ligand) is classified accordingly to its membranous topology as type II transmembrane protein that was identified by two research groups as a member of the TNF superfamily [1,2]. Unlike other TNF family members, such as TNFα or CD95L/FasL, TRAIL was detected in numerous normal as well as malignant cells and tissues [1,2]. In addition, it has been shown that TRAIL triggered apoptotic cell death in cancer cells, while most normal cells remained unaffected [3-7]. Interestingly, treatment of tumour cells with recombinant TRAIL suppressed their growth in mice [8] and improved survival of tumour-bearing animals [3]. Thus, making the TRAIL-system a promising target for anticancer therapies, that are still under investigation in several clinical trials as reviewed by Dimberg and colleagues [9].

The identification of five receptors that are capable to bind TRAIL suggests that signal transduction mediated by TRAIL is more complex when compared to the signaling pathways of other TNF-family members [7,10-16].

Upon binding of TRAIL to the death domain containing receptors TRAIL-R1/DR4 and TRAIL-R2/DR5/KILLER/TRICK2, receptor homotrimerization is induced, leading to the formation of a so called death inducing signaling complex (DISC) which sets the apoptotic machinery in motion [5,6,15-21]. However, TRAIL-R3/DcR1/TRID, lacking a cytoplasmic domain and TRAIL-R4/DcR2/TRUNDD, exhibiting an incomplete death domain, protect cells from TRAIL-induced cell death by direct antagonism [10,11,13-17,22]. Although with a low affinity, osteoprotegerin (OPG) has also been reported to bind TRAIL [12].

In addition to the apoptosis inducing signaling cascade, TRAIL can also activate pathways that promote survival and cell proliferation via the NF-кB, protein kinase B/Akt and MAP-Kinase pathways [10,23-27]. Thus, cells that are characterized by a death resistant phenotype when treated with TRAIL may convert into highly aggressive tumours that exhibit an increase in proliferation and metastatic capacity when stimulated with TRAIL [28,29]. Thereby, mechanisms promoting TRAIL resistance have been identified on different levels of the TRAIL pathway. These mechanisms include i.e. mutations of death receptors, overexpression of FLICE-like inhibitory protein, Bcl2 family proteins or Inhibitor of apoptosis (IAP) proteins [9].

Recently, several studies have focused on the prognostic significance of the TRAIL-system in different types of cancer. Interestingly, TRAIL-positive tumours less frequently obtained a pathological complete response in cervical cancer [30]. Poorly differentiated areas in NSCLC showed a strong staining pattern for TRAIL [31] and in colorectal cancer an increased TRAIL expression within the tumour was associated with worse overall survival [32].

Alternative splicing is a posttranscriptional modification process that has been shown to be involved in the regulation of apoptosis by promoting the translation of multiple proteins from a single gene that results in a sometimes functionally heterogenic pool of proteins with antagonistic functions [33,34].

Recently, we were able to identify and characterize also alternative splice variants of genes that belong to the TRAIL-system [35,36]. Alternative splicing of the TRAIL pre-mRNA led to the synthesis of two isoforms that were designated TRAIL-β and TRAIL-γ [35]. Both TRAIL-β and TRAIL-γ exhibited structurally an extensive truncation of their N-terminal binding domain which functionally was associated with the inability of mediating any proapoptotic signal.

Although previously the expression levels of TRAIL were investigated in gastric carcinomas [37], to date there are no data available that analyzed the expression of the alternative TRAIL splice variants in malignant diseases such as gastric carcinomas (for better discrimination TRAIL is denoted in this article as TRAIL-α). Thus, in our study we focused now on the quantification of mRNA levels of TRAIL-α TRAIL-β and TRAIL-γ in gastric carcinomas and correlated these expression levels with clinico-pathological variables and survival data. Since there are currently no specific antibodies available that can distinguish between the different splice variants, we quantified their expression levels by performing a reverse transcriptase and polymerase chain reaction (RT–PCR).

Using this experimental approach our data suggest that alternative splicing of the TRAIL gene might be involved in the molecular pathology of gastric carcinomas.

## Methods

### Patients and specimens

Matched neoplastic and non-neoplastic tissue specimens were obtained from 41 consecutive patients who underwent subtotal or total gastrectomy because of gastric carcinomas between 1996 and 2000.

After surgical resection, specimens of gastric carcinomas and corresponding normal gastric tissue were excised by a pathologist and immediately snap frozen in liquid nitrogen and stored at -80°C until usage. Tumour samples were selectively taken from the macroscopically identified tumour centre and the content of at least 70% of tumour was verified by microscopy. Pathological review, tumour staging, grading and typing were performed according to the principles outlined by the WHO [38] and the UICC [39]. Clinico-pathological parameters such as age, sex, primary site of disease, *Helicobacter pylori* infection, lymphatic vessel invasion and blood vessel invasion were recorded. Ethics Committee of the Medical Faculty, University Duesseldorf approved the study protocol (Number 3821).

### RNA isolation

Total RNA of tissue specimen were isolated by performing the acid guanidium thiocyanate phenol chloroform extraction with minor modifications as described previously [40]. The quality of total RNA was confirmed by integrity of the 28S and 18S ribosomal RNA in ethidium-bromide stained agarose gels.

### Reverse transcription and Semi-quantitative Polymerase chain reaction

Reverse transcriptase reaction was performed in a final volume of 20 μl using 2 μg total RNA and a final concentration of 5 mM MgCl_2_, 1 X reverse transcription buffer, 1 mM each dNTP, 20 U recombinant RNase inhibitor RNasin, 15 U AMV reverse trancriptase as well as 0.5 μg oligo(dT) primer (all Promega, Heidelberg, Germany). The samples were incubated at 42°C for 1 hour with a final denaturation at 94°C for 15 min.

The amplification of the TRAIL splice variants was performed on the LightCycler System (Roche Diagnostics, Mannheim, Germany) using the Platinum SYBR Green qPCR Super Mix-UDG kit (Invitrogen, Karlsruhe, Germany). Therefore, 4 μl of synthesized cDNA was amplified in a total volume of 20 μl containing 10 μl Platinum SYBR Green qPCR Super Mix-UDG which included the Taq DNA polymerase, SYBR Green I fluorescent dye, reaction buffer, dNTPs and uracil DNA glycosylase (UDG), 1 μl BSA and 10 pmol of each TRAIL-α specific primer (forward primer, GTC AGC TCG TTA GAA AGA TGA T and reverse primer, GCT CAG GAA TGA ATG CCC AC), TRAIL-β specific primer (forward primer, ATG GCT ATG ATG GAG GTC CA and reverse primer, GCT TTT CTT TCT AAC GAG CTG A); TRAIL-γ specific primer (forward primer, ATG GCT ATG ATG GAG GTC CA and reverse primer, GCT TTT CTG CTT CAG CTC GT). In addition, the housekeeping gene GAPDH was amplified using specific primers (forward primer, ACG GAT TTG GTC GTA TTG GGC G and reverse primer, CTC CTG GAA GAT GGT GAT GG).

PCR assays were composed of an initial denaturation at 95°C for 5 min, followed by 50 cycles of 1 s denaturation at 95°C, 20 s annealing at 64°C, extension at 72°C for 20 s at a temperature transition rate of 20°C/s and a final extension at 72°C for 5 min. In every PCR the hot start was performed to prevent the formation of unspecific products and primer dimers. In addition to the melting curve analysis, specificity of the PCR was controlled by agarose gel electrophoresis and products were sequenced with gene specific oligonucleotides.

### Quantification of TRAIL variants

The amount of TRAIL-α, TRAIL-β and TRAIL-γ transcripts was calculated semi-quantitatively in relation to the amount of the amplified housekeeping gene GAPDH serving as external standard. For the relative quantification, GAPDH specific PCR on cDNA standards comprising a fivefold dilution series on cDNA synthesized from HeLa cells was performed and used to create a standard curve by plotting the crossing point (CP) values against the dilution factor. Every dilution was run at least in duplicate. TRAIL-α, TRAIL-β, TRAIL-γ and GAPDH expression levels were normalised to the standard curve by GAPDH expression levels obtained from these cDNA standards which were amplified along the samples in every run. The number of gene copies was calculated by the LightCycler Software Version 3.5.3 according to the second derivative maximum method.

### Statistical analysis

GAPDH expression values for each sample were used to normalise the respective amplification values of TRAIL transcript and to calculate the ratios of relative mRNA levels. Statistical analysis was performed using SPSS 12.01 statistical software package (SPSS Inc., Chicago, IL, USA) or Graph Pad Prism 5 (GraphPad Software, Inc., La Jolla, CA, USA). To determine the differences of expression levels between the TRAIL splice variants in normal or neoplastic transformed gastric tissue statistical analyses were performed by using the Wilcoxon test.

The differences in the expression levels of all TRAIL variants according to clinico-pathological parameters and between gastric carcinomas and normal gastric tissues were analyzed using the Mann–Whitney–U–test. One-way ANOVA and Bonferroni's Multiple Comparison test was performed to compare expression levels of TRAIL splice variants with the primary site of gastric cancer. A two-tailed p-value less than 0.05 was considered to indicate statistical significance.

For survival analyses the median expression level was calculated and patients were categorized into two groups (≥ median and < median). Life table curves were estimated using the Kaplan-Meier analysis and the differences in survival were compared using the log-rank-test. A p-value less than 0.05 indicated statistical significance. Multiple proportional hazard Cox regression analysis was performed to investigate the prognostic value of TRAIL splice variants and clinico-pathological variables. The AIC (Akaike Information Criteria) was used for model selection.

## Results

### TRAIL splice variants are expressed in neoplastic and non-neoplastic gastric tissues

In this study we included patients that had undergone subtotal or total gastrectomy because of gastric carcinomas with UICC stages I-IV irrespectively of the tumour grading, histological type, margins of resection, age, sex or tumour localisation. Thus, we enrolled forty-one patients in this study comprising 27 male and 14 female patients with a median age of 64 ± 10.9 years (range 40–80 years). Samples included gastric carcinomas classified as UICC stage I (n = 11), stage II (n = 13), stage III (n = 9) and stage IV (n = 8) (Table 1). In seven patients margins of resection were tumour positive as diagnosed by histology and therefore being classified as R1 resection. Four patients showed at the time of diagnosis already distant metastases.

**Table 1:** Distribution of tumour staging and typing of the 41 gastric carcinomas

**pTNM stage**		**UICC stage**	**No. of patients**	
pT1			4	
	N0M0	I A		3
	N1M0	I B		1
pT2			28	
	N0M0	I B		7
	N1M0	II		13
	N2M0	III A		5
	N3M0	IV		3
pT3			8	
	N1M0	III A		3
	N2M0	III B		1
	N3M1	IV		4
pT4			1	
	N1M0	IV		1

**Figure 1:**
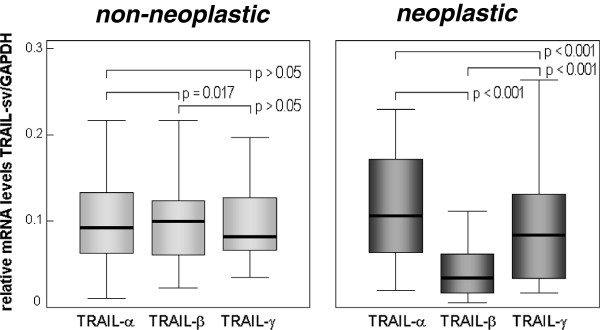
**Expression levels of TRAIL splice variants.** Relative (GAPDH-normalized) mRNA levels of the different TRAIL variants in non-neoplastic and corresponding neoplastic gastric tissue specimen (N = 41). P-values were calculated by the Wilcoxon-test; sv = splice variant.

Irrespective of the histological type, grade and stage of the tumour, TRAIL-α TRAIL-β and TRAIL-γ could be detected in all gastric carcinoma specimens and corresponding normal gastric tissue (Additional file 1: Figure S1). Specificity of amplification was revealed by gel electrophoresis which showed only a single amplification product (Additional file 1: Figure S1) as well as by melting curve analysis (Additional file 2: Figure S2). Whereas expression levels of the distinct TRAIL variants showed only minor differences in normal gastric tissues, TRAIL-α was the predominant transcript in gastric carcinomas (p < 0.001) (Figure 1).

### Correlations between the expression levels of TRAIL splice variants and clinico-pathological parameters

To further evaluate whether expression patterns of the different TRAIL variants might correlate with histo-pathological variables such as tumour staging, grading, typing, blood vessel invasion, lymphatic vessel invasion or *Helicobacter pylori* infection and other clinical parameters we performed the Mann–Whitney–U–test for nonparametric data or one-way ANOVA and Bonferroni's Multiple Comparison test to compare TRAIL expression with tumour location. As summarized in Table 2, this analysis did not provide any statistically significant correlation between the investigated clinico-pathological variables and the expression levels of wild type TRAIL-α or the alternative splice variants TRAIL-β and TRAIL-γ.

**Table 2:** Correlation between expression levels of TRAIL variants and clinicopathological parameters

		***p*-value**		
**Variables**	**No. of patients**	**TRAIL-α**	**TRAIL-β**	**TRAIL-γ**
*Primary site*		0.898	0.415	0.728
Proximal	16			
Corpus/Fundus	13			
Antrum/Pylorus	12			
*Stage (UICC)*	24	0.682	0.588	0.721
I + II	17			
III + IV				
*Histological grade*^a^				
G2	9	0.625	0.582	0.494
G3	31			
*Histological type*				
Intestinal	17	0.089	0.272	0.231
Diffuse	21			
Mixed	^b^			
*Lymphatic vessel invasion*				
Positive	23	0.948	0.509	0.895
Negative	18			
*Blood vessel invasion*				
Positive	8	0.349	0.633	0.681
Negative	33			
*Helicobacter pylori infection*				
Positive	13	0.234	0.127	0.071
Negative	28			
*Sex*				
female	14	0.945	0.290	0.858
male	27			
*Age*				
< 64 Jahre	19	0.764	0.140	0.969
≥ 64 Jahre	22			

^a^G1 (n=0) and G4 (n=1) not included due to the low case number.

^b^Not included in statistical analysis due to the low case number.

### TRAIL-β is downregulated in gastric carcinomas

**Figure 2:**
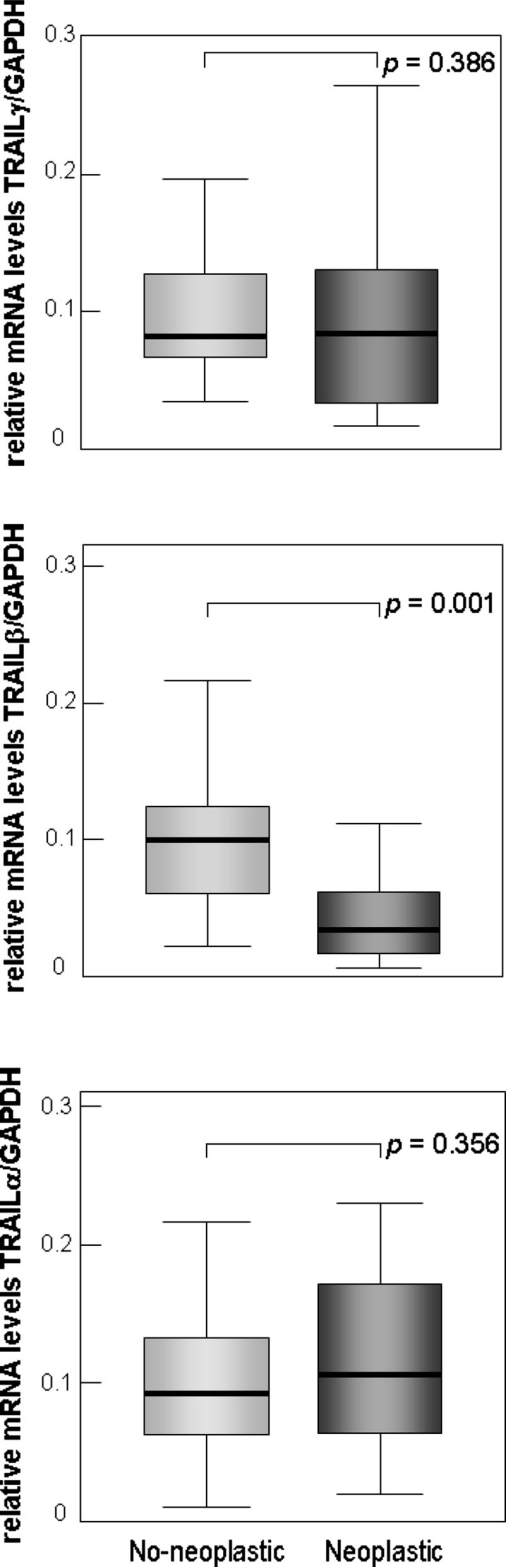
**Expression of TRAIL-β is downregulated in gastric carcinomas.** Paired samples (N = 41) of non-neoplastic and neoplastic gastric tissue where compared for expression levels of TRAIL-α, -β and -γ. Two-tailed P-values were calculated by the Mann–Whitney–U–test.

Recently, exclusively the expression of TRAIL without referring to its alternative splice variants has been analyzed in primary and metastatic gastric carcinomas. Therefore, we analyzed the expression of the different TRAIL splice variants by comparing matched neoplastic and non-neoplastic tissue samples. Whereas the expression levels of TRAIL-α and -γ showed no significant difference when comparing gastric carcinomas with the corresponding normal gastric tissue, TRAIL-β expression was significantly decreased in gastric carcinomas (p = 0.001) (Figure 2).

**Figure 3:**
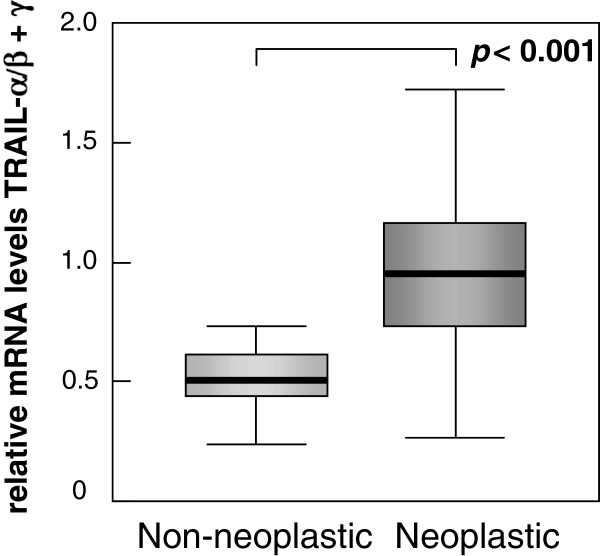
**Ratio of mRNA levels between proapoptotic and non-proapoptotic TRAIL isoforms**. A significant increase for proapototic TRAIL-α becomes evident in gastric carcinomas (N = 41) when comparing the proapoptotic TRAIL-α with the non-proapoptotic TRAIL-β and -γ isoforms.

Previously we reported that TRAIL-β and TRAIL-γ are characterized by the loss of their proapoptotic function. Thus, a reduced production of the non-proapoptotic isoforms by alternative splicing might result in an accumulation of proapoptotic TRAIL-α mRNAs. Therefore, we compared the mRNA expression levels of proapoptotic TRAIL-α with those of non-proapoptotic TRAIL-β and -γ in non-neoplastic as well as neoplastic tissues by calculating the ratio between mRNA levels of proapoptotic TRAIL-α on one site and non-proapoptotic TRAIL-β plus TRAIL-γ on the other site. Interestingly, this ratio revealed a significant 2-fold increase of proapoptotic TRAIL-α in gastric carcinomas when compared to normal tissue specimens (Figure 3).

### Prognostic value of non-proapoptotic TRAIL-γ mRNA

**Figure 4:**
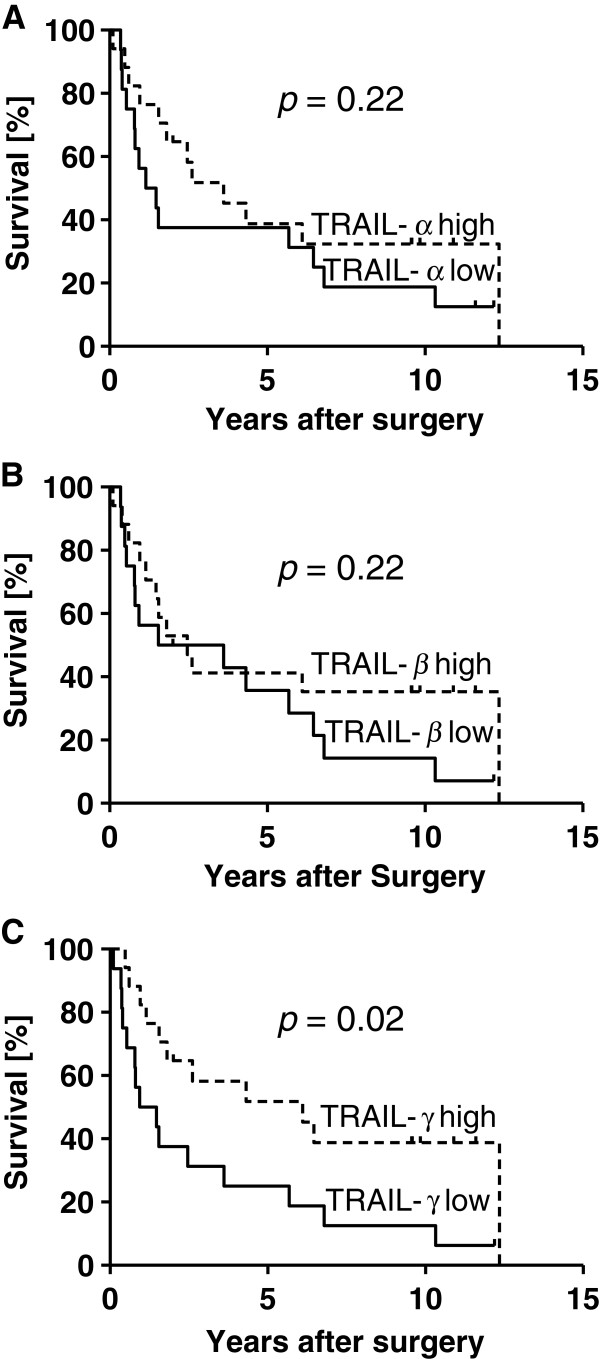
**Prognostic impact of TRAIL splice variants.** Kaplan-Meier survival curves for TRAIL-α **(A)**, TRAIL-β **(B)** and TRAIL-γ **(C)** calculated from 33 patients with gastric carcinomas. Expression of the non-proapoptotic TRAIL-γ variant was significantly associated with a higher survival.

In order to determine a prognostic value of mRNA expression levels of TRAIL splice variants in gastric carcinomas we estimated survival curves according to Kaplan-Meier. To eliminate any bias on survival analyses, eight patients were excluded from survival analyses because of distant metastases (n = 4) and/or non-tumour free resection margins (n = 7). The median postoperative follow up of the remaining patients (n =33) was 24 ± 51 months (range 1 – 148 months). Whereas no statistical difference in survival could be observed for the expression levels of TRAIL-α, and -β (Figure 4A and B), high expression of non-proapoptotic TRAIL-γ variant (≥ median 0.088) was significantly (p = 0.02) associated with a better survival (TRAIL-γ high vs. TRAIL-γ low: 6.1 vs. 1.2 years) (Figure 4C).

To provide better estimates of survival probabilities we explored the impact of several variables on survival using two Cox regression models. Thus, the model with all possible interactions (model 1) such as the level of expression of TRAIL-α, -β or -γ (high vs. low), lymphatic vessel invasion, blood vessel invasion, histological type, age, lymph node metastases, UICC stage and sex showed an AIC value that was comparable to the AIC value of the best model (model 2) (Table 3). In addition, as the analysis of variance (ANOVA) revealed, interactions among these variables were not statistically significant (p = 0.51). We conclude empirically that the three variables: expression level of TRAIL-γ, age, and UICC stage were independent prognostic factors.

**Table 3:** Multivariate survival analysis after best model selection by the AIC

	**Model 1**			**Model 2**		
**Variable**	**HR**	**95% CI**	***p*-value**	**HR**	**95% CI**	***p*-value**
TRAIL-α	1.353e-01	1.074e-11 – 1.705e+09				
TRAIL-β	1.934e+01	2.811e-23 –1.33331e+25				
TRAIL-γ	1.682e-07	8.462e-14 – 3.343e-01	*	2.595e-07	5.902e-12 – 0.01141	**
Histological type	2.183e+00	4.643e-01 – 1.026e+01				
Lymph node Metasasis	2.637e+00	3.606e-01 – 1.928e+01				
UICC	9.464e+00	1.609e+00 – 5.568e+01	*	1.757e+01	4.524e+00 – 68.2543	***
Age	1.133e+00	1.048e+00 – 1.225e+00	***	1.119e+00	1.048e+00 – 1.19422	***
Sex	1.111e+00	3.825e-01 – 3.225e+00				
R_square_		0.607			0.584	
AIC		104.3138			100.2167	

Significance codes: * indicates a *p*-value < 0.05, *** < 0.01 and *** < 0.001.

## Discussion

In this study, we analyzed the expression levels and prognostic value of TRAIL splice variants in gastric carcinomas and compared the expression of these variants in neoplastic tissues with the mRNA levels in corresponding normal gastric tissues. In contrast to the work by Koyama et al. [37] that reported a TRAIL protein expression in 55.6 % of the cells from primary gastric carcinomas and 53.7 % of the cells from metastatic gastric carcinomas, respectively, we were able to detect TRAIL in all gastric carcinoma samples on mRNA levels. This difference might be explained by the use of the more sensitive PCR method in our study. However, our study demonstrates for the first time the expression of the previously identified TRAIL isoforms (i.e. TRAIL-β and -γ) on mRNA levels in neoplastic and non-neoplastic gastric tissue specimens.

Recently, TRAIL has attracted a lot of attention as TNF-superfamily member because it is capable to trigger apoptosis in neoplastic transformed cells, implicating its potential as promising agent in targeted therapies against cancer [3-9]. Although in some cases the combination with chemotherapeutic agents is necessary to overcome TRAIL-resistance, first clinical trials show antitumour activity only with mild side effects [9]. Aside of its antitumor effects, TRAIL can activate via NF-кB signaling pathways that promote survival by activation of protein kinase B/Akt and MAP-Kinases [10,23-27].

However, the diversity in controlling TRAIL actions has been extended by the molecular cloning of TRAIL-β and -γ, which are structurally characterized by an extensive deletion of their N-terminal binding domain that consequently results in a loss of function when overexpressed in mammalian cells [35]. Thus, these alternative splice variants may be involved in the fine-tuning of TRAIL actions by simply influencing the translation of the classical apoptosis inducing TRAIL-α variant. In this context, alternative splicing of pre-mRNA was found to play a functionally important role as regulatory mechanism for apoptosis by processing distinct mRNAs from a pre-mRNA that in turn results in the translation of a pool of protein isoforms exhibiting often antagonistic biological properties [33].

Herein, we show that TRAIL-α and its alternatively spliced non-apoptotic isoforms TRAIL-β and -γ were detectable on mRNA levels in matched pairs of normal and neoplastic gastric carcinoma tissue specimens from patients that underwent surgery because of gastric carcinoma. Moreover, these TRAIL-variants were expressed in all histological types of gastric carcinoma, irrespective of tumour grading and staging. Interestingly, proapoptotic TRAIL-α exhibited the highest expression among the TRAIL variants in neoplastic tissues, but not in normal tissue specimens. In addition, the comparison between normal and tumour tissue illustrated a significant decrease of TRAIL-β mRNA levels in gastric carcinomas. A first clue for a functional role of this tumour specific downregulation became evident when comparing the expression levels of the proapoptotic TRAIL-variant with the non-proapototic isoforms (TARIL-β and -γ), showing a tumour specific increase of proapoptotic TRAIL-α. Thus, the tumour specific downregulation of the TRAIL-β mRNA might affect the TRAIL-α functions by a regulation on mRNA levels. Since all TRAIL variants originate from a common pool of a precursor RNA, the process of alternative splicing could permit an increase of proapoptotic TRAIL-α mRNA if for example the spliceosome catalyzes less RNAs with excluded exons such as TRAIL-β and TRAIL-γ transcripts. As a consequence, this could result in an increase of translated TRAIL-α protein which could support tumour growth by two ways. First, a TRAIL-induced activation of NF-кB could mediate the transcription of antiapoptotic targets such as XIAP and cFLIP, protecting the tumour from proapoptotic stimuli and permitting proliferation and metastasis. In this context, it has been demonstrated that TRAIL supported the conversion into a highly metastatic phenotype in pancreatic carcinomas by the induction of proinflammatory cytokines [29]. On the other hand, as suggested by others, the overexpression of ligands of the TNF-family such as FasL and TRAIL may be associated with immunological advantages for tumour cells by counterattacking tumour infiltrating cytotoxic lymphocytes [41-43]. In this context, a decrease in the processing of non-proapoptotic TRAIL variants (i.e. TRAIL-β and TRAIL-γ) as we observed in gastric carcinomas could permit the increase of translated TRAIL-α which in turn could induce programmed cell death in tumour infiltrating immune cells and thus promoting the immune escape and tumour surveillance of gastric carcinomas. Since a decrease of synthesized TRAIL-γ transcripts could permit an increase of transcribed and finally translated proapoptotic TRAIL-α, this could also explain our observation that downregulation of TRAIL-γ was associated with a worse prognosis. Thus, we hypothesize that the biological property of non-proapoptotic TRAIL splice variants has to be more likely interpreted as result of competing for translation with proapoptotic TRAIL-α.

## Conclusion

Taken together, our present study provides first evidence on the in vivo expression of TRAIL-splice variants in gastric carcinomas. The tumour-specific downregulation of TRAIL-β was associated with an increase of the proapoptotic TRAIL-α isoform in neoplastic tissues when compared to corresponding non-neoplastic tissue specimens. In addition, multivariate analyses identified TRAIL-γ as an independent prognostic marker. These data support the hypothesis that alternative splicing of TRAIL may play a crucial role in the tumour biology of gastric carcinomas.

## Competing interests

The authors declare that they have no competing interests.

## Authors’ contributions

AK and CM designed and coordinated the study, interpreted the data and wrote the manuscript. AK, SM and NW performed and analyzed the Semi-quantitative Polymerase chain reaction. SH and NHS contributed substantially to the data acquisition and drafting the manuscript. PEV and NHS contributed to and supervised the statistical analysis. CM and SH were involved in the sample acquisition, sample selection, clinical data acquisition and preparing the manuscript, JSAE, HEG, and WTK contributed to the acquisition and interpretation of the data and revised the manuscript critically. All authors read and approved the final version of the manuscript.

## Pre-publication history

The pre-publication history for this paper can be accessed here:

http://www.biomedcentral.com/1471-2407/13/384/prepub

## Supplementary Material

Additional file 1: Figure S1 Expression of TRAIL variants. RT-PCR analyses of TRAIL splice variants in gastric carcinoma **(A-C)** and normal gastric tissue **(D-F)** specimen by Real-Time-PCR (LightCycler) technology. SYBR Green I mediated fluorescence (y-axis) upon amplification was measured once per cycle (x-axis). TRAIL-α could be detected at cycles 24–28, whereas TRAIL-β and TRAIL-γ were detectable at cycles 28–32. The specificity of amplification products by agarose gel electrophoresis is shown in the upper framed box. Click here for file

Additional file 2: Figure S2 Melting curve analyses of amplified PCR products. Melting curve analyses from exemplary PCR experiments of **A)** TRAIL-α, **B)** TRAIL-β and **C)** TRAIL-γ from exemplary PCR experiments demonstrated that only one specific product was amplified. Click here for file
