# Supplementary figures and images for: Expression of TRAIL-splice variants in gastric carcinomas: identification of TRAIL-γ as a prognostic marker
Source: BMC Cancer. 2013 Aug 12;13:384. doi: 10.1186/1471-2407-13-384 (PMC3751299; doi:10.1186/1471-2407-13-384)

## Slide 1
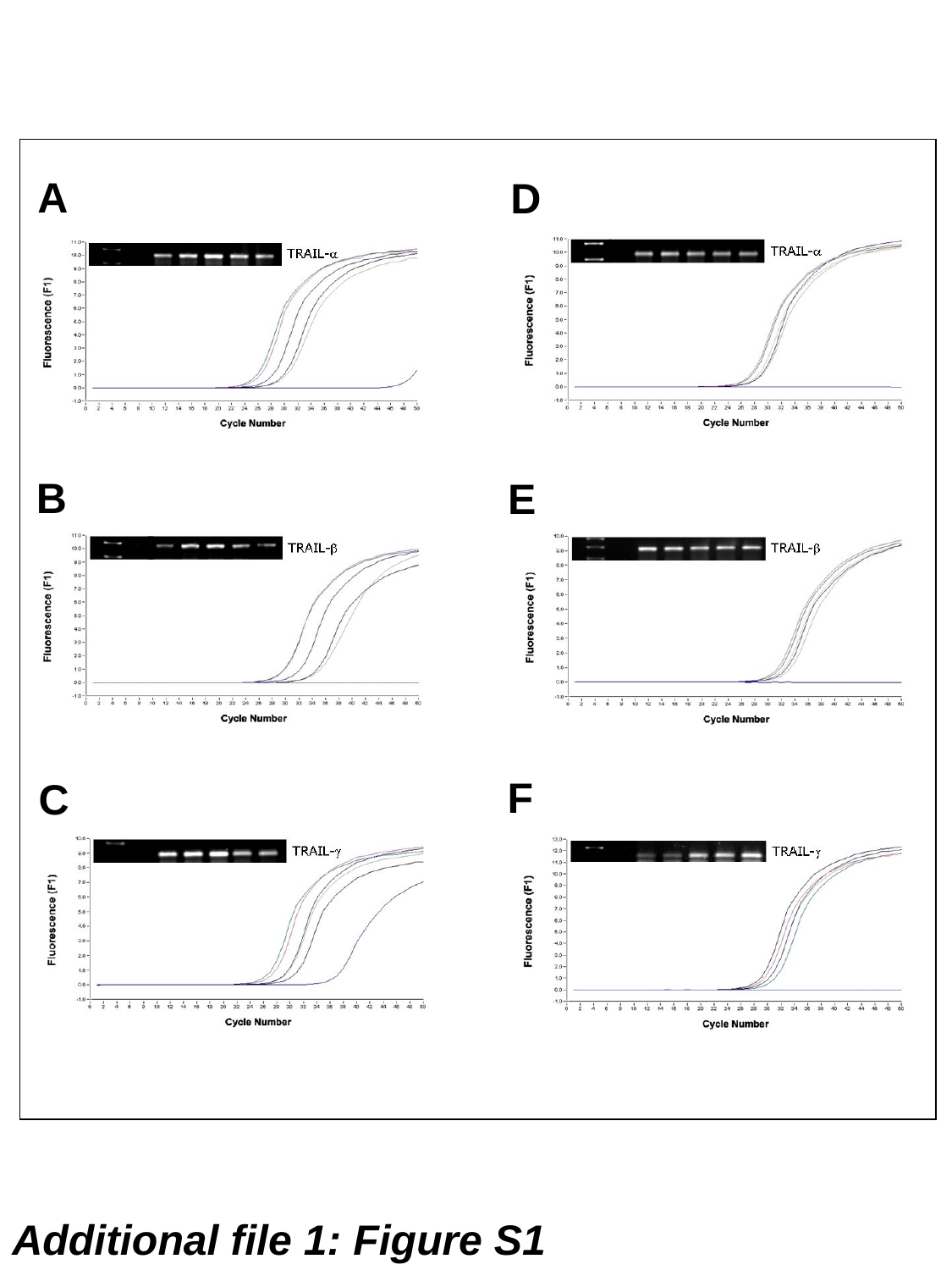

A
D
B
E
F
C
Additional file 1: Figure S1

Supplement: Additional file 1: Figure S1 — Expression of TRAIL variants. RT-PCR analyses of TRAIL splice variants in gastric carcinoma (A-C) and normal gastric tissue (D-F) specimen by Real-Time-PCR (LightCycler) technology. SYBR Green I mediated fluorescence (y-axis) upon amplification was measured once per cycle (x-axis). TRAIL-α could be detected at cycles 24–28, whereas TRAIL-β and TRAIL-γ were detectable at cycles 28–32. The specificity of amplification products by agarose gel electrophoresis is shown in the upper framed box. [file 1471-2407-13-384-S1.ppt]

## Slide 1
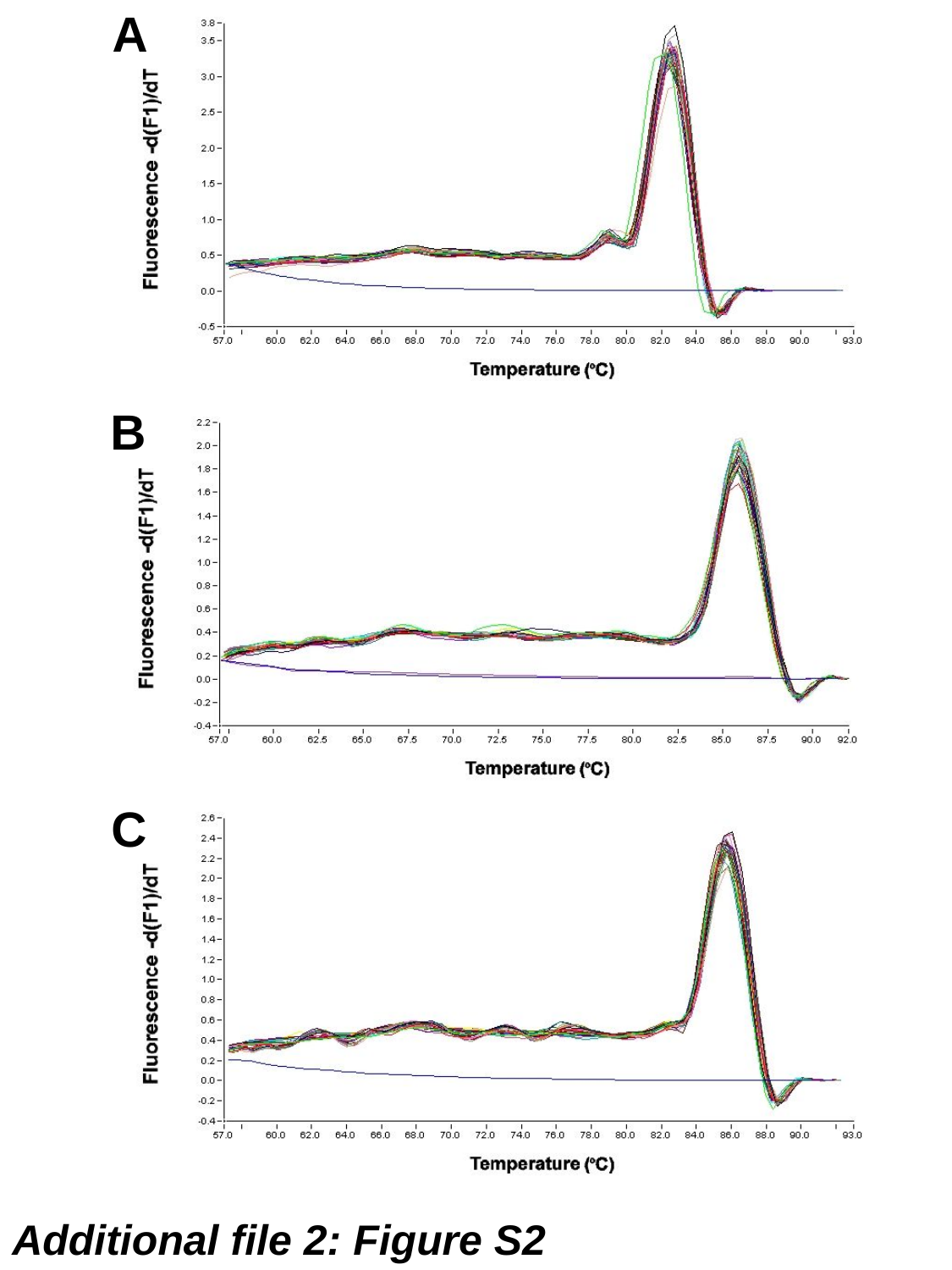

A
B
C
Additional file 2: Figure S2

Supplement: Additional file 2: Figure S2 — Melting curve analyses of amplified PCR products. Melting curve analyses from exemplary PCR experiments of A) TRAIL-α, B) TRAIL-β and C) TRAIL-γ from exemplary PCR experiments demonstrated that only one specific product was amplified. [file 1471-2407-13-384-S2.ppt]
